# Supplementary material for: Splice-Junction-Based Mapping of Alternative Isoforms in the Human Proteome
Source: Cell Rep. Author manuscript; Available in PMC 2020 Jan 15. (PMC6961840; doi:10.1016/j.celrep.2019.11.026)
Supplement: 2 [file NIHMS1546469-supplement-2.zip › DF1/PXD006675/PulmonaryArtery/PulmonaryArtery_1_CLTCL1_LQEHFQLQNLGINPANIGFSTLTMESDK.pdf]

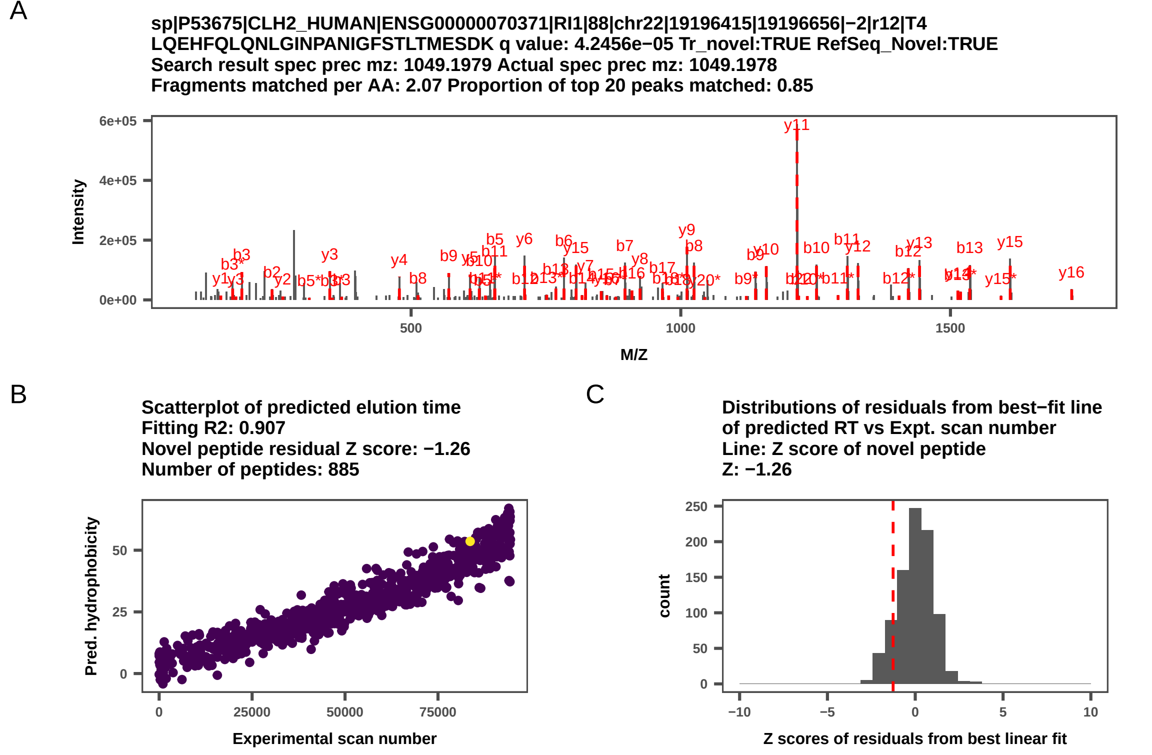
A
sp|P53675|CLH2_HUMAN|ENSG00000070371|RI1|88|chr22|19196415|19196656|−2|r12|T4
LQEHFQLQNLGINPANIGFSTLTMESDK q value: 4.2456e−05 Tr_novel:TRUE RefSeq_Novel:TRUE
Search result spec prec mz: 1049.1979 Actual spec prec mz: 1049.1978
Fragments matched per AA: 2.07 Proportion of top 20 peaks matched: 0.85
Intensity
6e+05
4e+05
2e+05
0e+00
500
1000
1500
M/Z
y11
y9
b8
y10
b10
b11
y12
y13
b12
b13
y15
y16
b5
y6
b6
y15
b7
y8
b3
b3*
y3
y4
b9
b8
b2
y2
B
Scatterplot of predicted elution time
Fitting R2: 0.907
Novel peptide residual Z score: −1.26
Number of peptides: 885
Pred. hydrophobicity
0
25
50
0
25000
50000
75000
Experimental scan number
C
Distributions of residuals from best−fit line
of predicted RT vs Expt. scan number
Line: Z score of novel peptide
Z: −1.26
count
0
50
100
150
200
250
−10
−5
0
5
10
Z scores of residuals from best linear fit
